# Maternal-fetal infections: Why do they matter?

**DOI:** 10.1080/21505594.2020.1759288

**Published:** 2020-05-03

**Authors:** Caroline Charlier, Marc Lecuit

**Affiliations:** Institut Pasteur, Biology of Infection Unit, Paris, France; Inserm U1117, Paris, France; French National Reference Center and WHO Collaborating Center for Listeria, Institut Pasteur, Paris, France; Necker-Enfants Malades University Hospital, Department of Infectious Diseases and Tropical Medicine, Institut Imagine, APHP; Université de Paris, Paris, France

The encounter of a pathogen and a pregnant host can happen at various steps of pregnancy and lead to a vast array of outcomes. Maternal infection can be more severe than in the general population, as reported for influenza [1], measles [2–4], dengue 5, or malaria [6]. Fulminant maternal infection may end pregnancy, as reported for Crimean-Congo hemorrhagic fever [7]. Some pathogens, such as *Listeria monocytogenes* and *Toxoplasma gondii*, are able to cross the placental barrier, replicate in the placenta and eventually infect the fetus [8,9]. The placental barrier may also be crossed at the time of delivery, and placental breaches allow maternal/fetal blood exchanges, leading to the vertical transmission of pathogens, such as Hepatitis B virus, and chikungunya virus [10]. At the fetal level, microbial tropism for fetal cells may account for specific teratogenic consequences, as recently evidenced by Zika virus-associated neuropathology [11]. The most successful medical intervention to tackle maternal-fetal infections and their dire consequences is the prevention of transmission, as controlling fetal infection once it is established is particularly challenging. In this respect, the history of how prevention of HIV congenital infection has been achieved is exemplary.

This “focus issue” aims at illustrating these issues with three major pathogens associated with maternal-fetal infections. Mysorekar *et al*. summarizes the scientific achievements achieved over the past 4 years regarding Zika virus, and present the current understanding of Zika’s neurotropism and its dramatic teratogenic consequences [11–14]. We detail the epidemiological and clinical features of maternal-fetal listeriosis and present its pathophysiology [8,9,15,16]. Finally, Blanche reviews the stunning achievements that led to the control of congenital HIV, which started in the mid-nineties when zidovudine was proven effective in reducing vertical infection, and continues with the ongoing efforts in screening and organizing the care of pregnant women at a global scale [17].

We hope that this “focus issue” which provides the readers with a targeted overview of maternal-fetal infections will inspire clinicians and scientists interested in this important field to synergize the medical and scientific dimensions of their research.
